# The Healthcare Systems Research Network (HCSRN) as an Environment for Dissemination and Implementation Research: A Case Study of Developing a Multi-Site Research Study in Precision Medicine

**DOI:** 10.5334/egems.283

**Published:** 2019-04-12

**Authors:** Alanna Kulchak Rahm, Ilene Ladd, Andrea N. Burnett-Hartman, Mara M. Epstein, Jan T. Lowery, Christine Y. Lu, Pamala A. Pawloski, Ravi N. Sharaf, Su-Ying Liang, Jessica Ezzell Hunter

**Affiliations:** 1 Geisinger, US; 2 Kaiser Permanente Colorado, Institute for Health Research, US; 3 The Meyers Primary Care Institute and Department of Medicine, University of Massachusetts Medical School, US; 4 University of Colorado, US; 5 Department of Population Medicine, Harvard Medical School and Harvard Pilgrim Health Care Institute, US; 6 HealthPartners Institute, US; 7 Weill Cornell Medicine, US; 8 Palo Alto Medical Foundation Research Institute, US; 9 Kaiser Permanente Northwest, US

**Keywords:** HCSRN, Lynch syndrome, Dissemination and implementation, implementation science, precision medicine

## Abstract

**Context:**

In existence for nearly 25 years, the Healthcare Systems Research Network (HCSRN) is an established and sustainable network of health care systems that serves as a “real world” laboratory to enable the integration of research findings into practice. The objective of this paper is to demonstrate how the HCSRN serves as an ideal environment for studying dissemination and implementation of evidence-based practices into health care systems through the example of developing a multi-site study on the implementation of evidence-based precision medicine practices.

**Case description:**

The “Implementing Universal Lynch Syndrome Screening (IMPULSS)” study (NIH R01CA211723) involves seven HCSRN health care systems and two external health care systems. The IMPULSS study will describe and explain organizational variability around Lynch syndrome (LS) screening to identify which factors in different organizational contexts are important for successful implementation of LS screening programs and will create a toolkit to facilitate organizational decision making around implementation and improvement of precision medicine programs in health care systems.

**Major Themes:**

The strengths of the HCSRN that facilitate D&I research include: 1) a culture of collaboration, 2) standardization of data and processes across systems, and 3) researchers embedded in diverse health care systems. We describe how these strengths contributed to developing the IMPULSS study.

**Conclusion:**

Given the importance of conducting research in real world settings to improve patient outcomes, the unique strengths of the HCSRN are of vital importance. The IMPULSS study is one case example of how the strengths of the HCSRN make it an excellent environment for research on implementing evidence-based precision medicine practices in health care systems.

## Context

Established in 1994, the Healthcare Systems Research Network (HCSRN) is a network of over 1,900 researchers across 19 health care systems who regularly collaborate and serve as a research laboratory based in “real-world” health care populations and environments and to enable the rapid integration of research findings into practice [1]. In this paper, we present a case study that leverages the strengths of the HCSRN for dissemination and implementation science research. The objective of this paper is to demonstrate how the HCSRN serves as an ideal environment for studying dissemination and implementation of evidence-based practices into health care systems through the example of developing a multi-site study on the implementation of evidence-based precision medicine practices into diverse health care systems.

### Definition of Dissemination and Implementation

The implementation of effective technologies and evidence-based precision medicine strategies into clinical practice is challenging and slow and contributes to variability and deficiencies in quality of care [2,3]. Contextual factors, such as organization mission and structure, economic considerations, provider preferences and readiness, as well as patient population and attitudes, influence implementation decisions within individual health care systems [4,5,6].

Dissemination and implementation (D&I) science bridges the gap between research and clinical practice by generating evidence to: 1) understand how evidence spreads to different stakeholders in the health care system (dissemination) and 2) characterize how the behavior and perspectives of clinical stakeholders, health care organizations, and patients influence the adoption, adaptation, and sustainability of evidence-based practices in real world settings (implementation) [4,7].

### The HCSRN for D&I Research

The HCSRN provides an optimal environment to conduct D&I studies. The HCSRN will celebrate its 25^th^ year of embedded research in health care systems in 2019; therefore is also an established and sustainable network in which to conduct D&I research. The “real-world” health care organizational infrastructure, clinical infrastructure, providers, and patient populations within the HCSRN represent diverse contextual factors to better understand the factors influencing dissemination and implementation of evidence-based practices.

**Figure 1:**
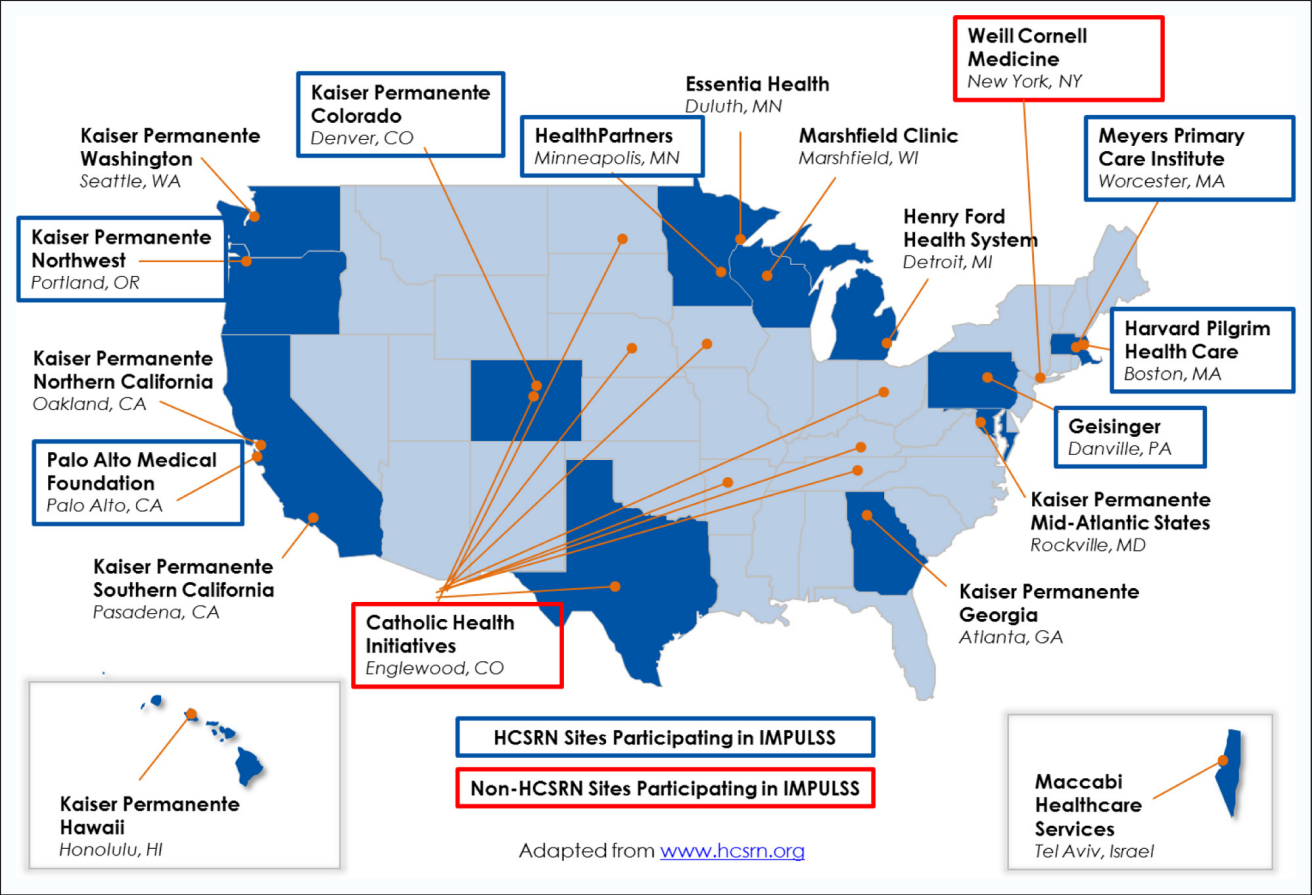
Map of HCSRN with IMPULSS Study Sites Highlighted.

Overall, the HCSRN member systems provide care for over 28 million individuals based in diverse geographic regions across 13 states (Figure 1). All HCSRN health care systems have robust electronic health record (EHR) systems as well as researchers embedded within the health care system and experienced in utilizing and interpreting the extensive EHR data. HCSRN systems have adopted a common data model, standardized administrative processes and procedures for collaborations with external researchers and organizations, as well as templates and best practices for data use agreements (DUAs) and for institutional review board (IRB) ceding to a single institution in order to facilitate multi-site research more efficiently and effectively.

In this paper, we present the case of developing a research study that leverages the HCSRN for D&I research in precision medicine. This multi-site project is a collaboration between seven systems across the HCSRN and two external organizations whose collaboration in the project was facilitated through the HCSRN. Due in part to the strengths of the HCSRN for D&I research, this project was subsequently funded by the National Institutes of Health (NIH).

## Case description: developing a research project in the HCSRN for D&I research

Benefits of conducting D&I science within the HCSRN can be illustrated through the example of the newly-funded study, “Implementing Universal Lynch Syndrome Screening (IMPULSS)” [8]. The IMPULSS study is a multi-site R01 that was submitted under the Program Announcement for Dissemination and Implementation in Health (DIRH) (PAR-18-007) and subsequently funded in July, 2017 by the National Institutes of Health (NIH) through the Beau Biden Cancer Moonshot funds (NIH R01CA211723).

The health care systems participating in IMPULSS were identified through collaborations facilitated by the HCSRN, the HCSRN Genomics Scientific Interest Group (SIG), and the Cancer Research Network (CRN). Seven HCSRN health care systems are participating in the IMPULSS study (Geisinger, Kaiser Permanente Northwest (KPNW), KP Colorado (KPCO), HealthPartners, Harvard Pilgrim Health Care, Sutter Health-Palo Alto Medical Foundation, and Meyers Primary Care Institute). Two non-HCSRN organizations (Catholic Health Initiatives and Weill Cornell Medicine) are also collaborating in the IMPULSS study (Figure 1).

Both non-HCSRN organizations were identified through existing collaborations with the HCSRN: the IMPULSS site principal investigator (PI) from Weill Cornell Medicine has an established record of collaboration within the HCSRN related to Lynch Syndrome through the CRN Scholar program [9]. An IMPULSS investigator associated with Catholic Health Initiatives was an active participant in the HCSRN Genomics SIG, through which the IMPULSS study was created.

Preliminary data for the IMPULSS project were gathered by surveying the two non-HCSRN organizations and all HCSRN organizations about their respective Lynch Syndrome screening practices in general. The application for funding was developed through a collaborative process which included the overall PI presenting the IMPULSS project concept in multiple HCSRN venues to identify researchers interested in collaboration. The administrative requirements (sub-award budgets, facilities and infrastructure, and planned enrollment tables) were efficiently facilitated through processes developed through the long-standing collaborations within the HCSRN to gather and share such materials for grant applications. This familiarity of administrative processes enabled the application process to run efficiently with the seven HCSRN sites so that administrative staff at the prime institution could focus on any procedural needs that arose with the non-HCSRN sites.

### Lynch Syndrome Screening and Contextual Differences Facilitating D&I Research

Lynch Syndrome (LS) is the most common form of inherited colorectal cancer and is also associated with significant risk for endometrial, ovarian, gastric, small bowel, and renal cancers, among others [10,11]. LS is characterized by mutations in mismatch repair genes and is inherited in an autosomal dominant pattern. Estimates indicate about 1 million people in the United States have LS [12,13]. LS accounts for 3–5 percent of all newly diagnosed CRC [10]; yet only about 2 percent of such individuals are identified as having the condition [13]. Universal Lynch syndrome screening is the systematic screening of all newly diagnosed cases of colorectal and endometrial cancers to identify patients whose cancer is related to LS [14]. LS screening includes evaluating tumors for mismatch repair gene deficiency and offering genetic counseling and confirmatory germline genetic testing to individuals who screen positive. LS screening is one of the first cost-effective [15,16] genomic medicine interventions with evidence [17] for saving lives and improving quality of life [18]. LS screening was first recommended by the Evaluation of Genetic Application in Practice and Prevention (EGAPP) working group in 2009 [14,19], is currently recommended by multiple professional organizations [10,20,21,22,23,24], is endorsed by the National Comprehensive Cancer Network (NCCN) [25], the Centers for Disease Control (CDC), and is an objective of the Healthy People 2020 initiative [26]. In September 2016, the Blue Ribbon Panel Report recommended LS screening as a high priority intervention with the potential to achieve the goals of the Cancer Moonshot [12].

Implementing LS screening in health care systems, however, involves multiple stakeholders and customization to local contextual factors, including integrating LS screening into individual organizational processes, communicating with different patient populations, and determining the costs of LS screening to systems and patients [27,28]. Because LS screening has infrequently and inconsistently been implemented across health care settings, there is poor understanding of how these contextual factors impede or facilitate implementation of such precision medicine programs in health care systems, and under what circumstances [29]. The unique contextual properties of the sites in the HCSRN created the opportunity to develop the IMPULSS study to contribute to this understanding.

Capitalizing on the variability and complexity of individual organizational structures and differences in the implementation of LS screening programs across health care systems, the IMPULSS study will describe and explain individual organizational variability. As shown in Table 1, some participating health care systems have implemented programs to screen all colorectal cancer (CRC) and endometrial cancer (EC) tumors diagnosed in the system as recommended by current guidelines, while others limit to CRC tumors only. Despite the evidence, multiple systems have yet to implement any program to systematically screen tumors for LS. This variability across systems will facilitate the identification of factors that enable or impede implementation in different organizational contexts. Information gained from this study will be used to create a toolkit to guide organizational decision making around implementation and facilitate implementation of LS screening and other precision medicine programs in health care systems broadly.

**Table 1:** Characteristics of Health Care Systems Participating in the IMPULSS Study and Variability in Implementation of Programs to Systematically Screen for Lynch Syndrome.

Health Care System	System Type	HCSRN Participant	LS Screening Implementation

Geisinger	Member and FFS	Yes	All CRC and EC
Sutter Health Palo Alto Medical Foundation	Member and FFS	Yes	No Program
Kaiser Permanente Colorado	Member only	Yes	No Program
Kaiser Permanente Northwest	Member only	Yes	All CRC and EC
Meyers Primary Care	Member and FFS	Yes	All CRC
HealthPartners	Member and FFS	Yes	All CRC and EC
Harvard Pilgrim	Member and FFS	Yes	All CRC
Weill Cornell Medical Center	FFS only	No	All CRC and EC
Catholic Health Initiatives	FFS only	No	Variable depending on Hospital location

FFS: Fee for Service; CRC: Colorectal Cancer; EC: Endometrial Cancer.

## Major Strengths of the HCSRN for D&I Research

The HCSRN is a unique environment for studying the dissemination and implementation of evidence-based practices, such as LS screening, because of the number of health care systems in various stages of implementing programs to adhere to guidelines. Here we further describe the strengths of the HCSRN for D&I through the themes of 1) a culture of collaboration over more than twenty years [30,31], 2) standardization of data and processes across systems [30,32], and 3) researchers embedded in diverse health care systems [1,30]. These strengths of the HCSRN are what made it possible to develop and fund the IMPULSS project. Below, we describe how each of these major strengths contribute to the IMPULSS study and D&I research in general (Table 2).

**Table 2:** The Major Strengths of the HCSRN and Benefits to D&I Research.

HCSRN Strengths	Example Benefit to D&I

Culture of Collaboration	An atmosphere of shared purpose across all staff levels facilitates research processes Provides opportunity to adapt processes and interventions implemented at one site to other sites and populations Leverages methodologic expertise at different sites
Standardization of data and processes across systems	A common data model streamlines analyses Standards for data storage and guidelines for addition of new data facilitate efficient cross-site collection and analyses Established templates for DUAs, IRBs and other research agreements allow efficient study management
Researchers embedded in diverse health care systems	Embedding researchers in real-world settings facilitates meaningful D&I research

### Culture of collaboration and leveraging methodological expertise

The HCSRN has a long-standing culture of collaboration at the organization, researcher, staff, and research methodology levels [30]. Over the past 25 years, member organizations have developed a trust and familiarity that facilitates efficient and effective communication between organizational research administrative staff. Individual researchers have established collaborations over this time period across a myriad of research areas to create ongoing research network programs and individual research projects [30]. The HCSRN also hosts annual research meetings for sharing research findings and lesson learned from one another [31]. The previously mentioned SIGs, including the Genomics SIG, meet regularly (http://www.hcsrn.org/en/Collaboration/SIG/) to foster collaboration and facilitate project development between HCSRN annual research meetings. There are also qualitative, quantitative, and mixed-methods experts collaborating across the HCSRN, as well as senior scientists collaborating to mentor junior researchers through formal and informal programs [9,31]. Specific to the IMPULSS study, the PI had early-career investigator status and utilized multiple mentors and senior collaborators within the HCSRN to assist in the process through study design, application writing, and the conduct of the project.

**Collaboration to build on prior studies:** The collaborative culture facilitates the ability of researchers to build on studies conducted at one site to inform and contribute to the design of subsequent studies at other sites. This is crucial in D&I research to facilitate the adoption of evidence-based practices. For example, a prior study of LS screening implementation at KPNW assessed patient and provider perspectives of LS screening but was limited to individuals newly diagnosed with CRC who had agreed to undergo screening for LS as part of a study protocol at a single site [33,34]. The IMPULSS study was designed to expand these prior results by exploring attitudes of organization stakeholders and patients with CRC from health care systems that are and are not screening for LS as part of routine clinical care; rather than being limited to understanding LS screening only from those patients and providers participating in a study protocol.

**Methodologic expertise and collaboration:** The robust sharing of research methodology and experiences that has been established over time in the HCSRN also enables leveraging expertise and institutional memory of the researchers and staff at various sites in the development of new studies within the real-world environment of the health care systems. Multiple prior HCSRN studies established the methodology proposed in the IMPULSS study to conduct key organizational stakeholder interviews across all participating systems centrally from one site (Geisinger) [35,36,37]. Methodological expertise within the HCSRN is further leveraged for the IMPULSS study as qualitative interviews of patients are being conducted centrally for all health care systems from another study location (KPNW) with a robust qualitative core and staff experienced at conducting such centralized patient interviews and utilizing well-organized processes for tracking and sending incentives [38]. Capitalizing on this expertise to propose centralized interviewing facilitates efficient use of study resources and enables a study of this size to be proposed and conducted.

### Standardization of data and processes across systems

The longevity of the HCSRN provides a wealth of experience in the understanding of data infrastructures and reliability and has resulted in robust streamlining of research administrative processes over time. Specific to IMPULSS, this familiarity with data infrastructure and existing administrative protocols are another strength of the HCSRN that made developing a study of this size possible within the limited research resources available in a funded study.

**Data standardization:** The common data model utilized by the HCSRN is the Virtual Data Warehouse (VDW) [32]. The VDW incorporates several domains related to health care utilization, including demographics, physical measures, personal medical history, surgical history, laboratory tests, procedures, prescription medications, diagnoses, and health plan claims [39]. For data that are not in the VDW, the collaborative environment of the HCSRN benefits projects that require new data elements. In general, genetic data and data related to tumor screening results and other parameters important to LS are not easily extracted from electronic data. For the IMPULSS study, multiple site PIs have been working to determine how to pull laboratory results pertinent to the tumor screening in LS as part of other research. These IMPULSS team members also developed approaches to identify newly diagnosed colon cancer, an important study population in IMPULSS. These processes leverage site-specific strengths and resources (VDW, tumor registries, or claim-based algorithms) to identify newly diagnosed cases of colorectal cancer patients, while achieving comparable analytic samples and empirical metrics across sites without each site expending resources to develop and validate these algorithms independently. The data parameters required to identify these patients will be used by all participating health care systems in IMPULSS to ensure standardization in eligibility criteria and contribute to efficiency in patient recruitment by facilitating electronic identification of eligible patients.

**Administrative process streamlining:** Over the years, the HCSRN has worked diligently to streamline administrative processes by developing and adopting pre-negotiated sub-award and DUA templates, as well as constructing standardized IRB templates. These data and organizational standardizations have made it possible to execute DUAs across the 7 HCSRN sites and the 2 non-HCSRN sites in the first year of the IMPULSS project. For IRB processes and to facilitate movement towards the single IRB process, standard operating procedures (SOPs) for ceding and an Inter-institutional authorization agreement (IIA) template for ceding within the HCSRN have existed for some time [30]. Within the IMPULSS study, eight sites chose to cede IRB oversight to Geisinger, the lead site. The remaining HCSRN site that did not cede was still able to efficiently utilize the Geisinger IRB application to create site-specific IRB documents due to the use of standardized IRB templates. The use of HCSRN-approved language for the DUAs and IRB templates provided a common starting ground to facilitate study work and recruitment in the first year rather than expending valuable study staff time and resources negotiating nine contracts and waiting for nine IRB reviews.

Standardization of data and processes are happening elsewhere and single IRB for multi-site projects funded by the NIH is required for projects funded after January 25, 2018 [40]. The HCSRN administrative processes and IRB templates for ceding predate the NIH single IRB requirements, giving the HCSRN participating sites more experience in conducting studies under a single IRB. This also facilitates the process for including non-HCSRN sites in studies or for HCSRN sites participating in studies with a non-HCSRN prime site responsible for all IRB administration.

### Researchers embedded in diverse health care systems

HCSRN organizations have researchers who are embedded within their respective health care systems and conduct research in “real-world” settings critically important to D&I research. These researchers have access to and established collaborations with their local clinical enterprises and a deep understanding of the contextual complexity of their organization’s data, business model, and organizational and leadership structure.

D&I research in general and the IMPULSS study specifically seeks to understand organizational complexity around implementation of evidence-based recommendations. For IMPULSS, the embedded researchers (the site PIs) were able to quickly assess specifics related to their organization’s process (or lack thereof) towards implementing universal tumor screening for LS (see above Table 1 – Lynch screening implementation column). This allowed us to develop and submit for funding the IMPULSS research project which will significantly contribute to implementation science by studying how different organizational contexts contribute to the complexity of implementing evidence-based precision medicine programs like LS screening.

Another challenge with D&I studies is that health care systems are rarely static. Because HCSRN investigators are embedded in their health care systems, they can notify the larger research team when changes that may impact the study happen. Based on experience from other implementation studies conducted by the PI [35,41], the IMPULSS study is designed to keep track of changes impacting LS screening at the external, system, provider, patient, and program levels (Table 3).

**Table 3:** Example of Changes by Domain That May Impact LS Screening at IMPULSS Study Health care Systems.

External Level	System Level	Provider Level	Patient Level	Program Level

Guideline changes	Mission changes	Knowledge change	Knowledge change	Protocol changes
New evidence	Leadership changes	Belief change	Expectation change	Cost changes
Payor policy changes	Organization changes	Readiness change	Population changes	Implementing program
New technology	New resources	Champion changes		Discontinuing program

For IMPULSS, a database is used to track changes to LS screening programs specifically and to clinical and organizational leadership for each health care system within the IMPULSS study. Information is entered into a database as reported by site PIs during regular study meetings. Data corresponding to change over time will contribute to study results as we identify those contextual factors needed for implementing effective LS screening and other precision medicine programs.

## Conclusions

Given the importance of conducting research in real world settings to improve patient outcomes, the unique strengths of the HCSRN are relevant now more than ever. The development and successful funding of the IMPULSS study is one case example of how the strengths of the HCSRN make it an important environment for research on implementing evidence-based practices in health care systems. Key strengths of the HCSRN important to D&I research include a culture of collaboration with high levels of productivity and experience, standardization of data collection and research procedures across systems, and researchers embedded in diverse health care systems. These strengths individually are not unique to the HCSRN; and in fact, are important to conducting D&I research in any network. Based on the case example of the newly-funded IMPULSS precision medicine study, we demonstrate it is the combination of these elements – collaboration, standardized data and processes – and embedded researchers that make the HCSRN one such environment to successfully design and conduct D&I research.
